# A Cremation Elegy (Poetical)

**Published:** 1874-05

**Authors:** 


### Our Exchanges.

### A CREMATION ELEGY.

*De mortuis not even bones.*

*"Where is thy grave, my love, I want to weep?"*

The chimney-stack through which my love has flown
  Be sacred now with all perpetual fires;
Her ashes, shrouded in this hollow urn,
  Are all that's left of dead love's fond desires.

How oft when wandering near this hallowed spot,
  And thinking of the days that are no more,
My yearning eyes shall seek yon chimney-pot,
  Thro' which sweet Ellen's volatiles did pour.

Sweet caborn, loved ammonia, water fine,
  HO, CO, NH, of daintiest mould,
And all she was; ah! wherefore then repine,
  Since not for aye the chemic forces hold?

Ye powers that hold above the silent dead
  Your everlasting speechless watch and ward,
That thaw the frost that binds so dear a head,
  That melt the golden bowl, the silver cord;

To you, ye viewless spirits of the air,
  Cohesion, chemical affinity,
Straight have I yielded up my Ellen fair
  To act upon her sweet divinity.

And you, my brothers, when my days are ended,
  I pray you this favor for me do,
Ah! lay my body there, whence she ascended,
  To fly up through the flue up which she flew.

